# Professional responsibility in obstetric-gynecological nursing: Update of the case law of the High Court of Justice of Andalusia (Spain)

**DOI:** 10.1371/journal.pone.0291669

**Published:** 2023-09-26

**Authors:** Luz Marina González-Hernández, Jorge San José

**Affiliations:** 1 Nurse Specialist in Obstetrics and Gynecology, University Master’s Degree in Nursing Direction and Management, Graduate in Nursing of the University of Seville, Seville, Spain; 2 Faculty of Health Sciences, Department of Health Sciences, Universidad Europea de Valencia, Valencia, Spain; University of Macerata: Universita degli Studi di Macerata, ITALY

## Abstract

**Background:**

Obstetric-Gynecological Nursing is articulated as one of the specialities with the greatest responsibility in the field of health care, due to the involvement of being in care not only for the life of the pregnant woman, but also for the future neonate. Settling down as a profession with a high number of legal claims, there are not many studies in Spain on legal claims in the field of Nursing in general, and Obstetric-Gynecological Nursing in particular.

**Methods:**

Retrospective analysis of judgments against midwives in the period from 2010 to the present through the CENDOJ database, with the aim of searching for criminal, civil and contentious-administrative judgments. Quality was assessed using the STROBE critical appraisal tool for observational studies.

**Results:**

19 judgments were selected from the 197 found that were related to the objective of the study. Fifty-three percent of the judgment analyzed resulted in acquittals, while the remaining 47% were upheld to varying degrees. Most of them were motivated damage to the newborn (79%), processed entirely through the contentious-administrative route.

**Conclusions:**

Most of the legal claims in the field of Obstetric-Gynecological Nursing are related to adverse events with fetal damage, most of them receiving higher monetary compensation as the contentious-administrative route is the jurisdiction with the highest number of claims filed, due to breaches of the *lex artis ad hoc*.

## Rationale of the study

Stillbirth-related neonatal death is one of the leading causes of mortality, particularly in low-income countries and is closely related to a high burden of stillbirths. Studies conducted by the Maternal and Child Epidemiology Estimation group (MCEE) [1] in 2013, found that neonatal deaths related to childbirth were the third leading cause of death in children under 5 years old.

The relevance of this work responds to the need for efficient organizational management models, based not only on specialization, but on continuous improvement, incorporating interpretations and arguments in which, from a legal point of view, the requirements for the proper performance of the professional activity of Obstetric-Gynecological Nursing are based.

Obstetrics and Gynecology is one of the specialties with the highest number of lawsuits, ranking 5th on the list of medical specialties with the highest number of demands in the 2020 annual statistics [2]. Therefore, it is essential for the collective that integrates this specialty and by extension for nursing in general, to know the characteristics and consequences of such claims.

Therefore, these professionals should know the legal implications of their activities, as well as the importance of judicial judgements and their application for the improvement of clinical and organizational protocols.

## Introduction

In the Spanish sphere, the training of the nurse specializing in obstetrics and gynecology is regulated in the Order SAS/1349/2009 [3] it is recognized as an essential professional in the social sphere of maternity and the comprehensive care of women throughout their sexual, reproductive and climatic life. The scope of action of the midwives covers the fields of Primary Care (health centers, work with the community, training in institutes…) and Specialized Care (hospital and centers attached to it).

The competency profile according to article 55 of Royal Decree 1837/2008 of 8 November, whose professional activities are, among others, the following [4,5] (Table 1).

**Table 1:** Competency profile of Midwife in healthcare area.

a. Provide care and assistance to the mother during childbirth and monitor the condition of the fetus in the womb through appropriate clinical and technical methods.
b. To take care of the normal delivery, when it is a vertex presentation, including, if necessary, the episiotomy and, in case of emergency, to attend the delivery in breech presentation.
c. Recognize in the mother or in the child the signs of abnormalities that require the intervention of a doctor and, where appropriate, assist him; take the necessary steps in the absence of the doctor, in particular the manual removal of the placenta, followed, where appropriate, by manual examination of the uterus.
d. Recognize and care for the newborn; take all the necessary initiatives and practice, if necessary, immediate resuscitation.
e. Assist and monitor the mother’s progress after childbirth and provide the necessary counselling regarding childcare so that she can ensure the optimal progress of the newborn.

This implies exercising a clinical judgment and scientific attitude to provide comprehensive care to women in all facets of their sexual, maternal and reproductive life by promoting the promotion and prevention of health, as well as the preservation of the mother-child binomial from the moment of birth to the 28 days of life of the newborn [6].

Placing ourselves in the analysis during the intrapartum process of the fetal frequency and the uterine dynamics through cardiotocography records (CTG) and the partogram, both are considered as tools that allow to evaluate changes in the pattern of normality. These elements of judgment constitute documents of great value because it allows to establish causal and temporal relationships [7,8]. Which means, it is a civil and criminal liability for the midwife.

Civil liability is understood as the "incorrect conduct, not adapted to the *lex artis*, which seeks compensation for damage caused by pecuniary means" [9,10], being a contractual and a non-contractual one. The first involves a breach of contract by a healthcare provider, regulated by the Spanish Civil Code in articles 1.101 and SS., which requires a bond and the element of guilt (fraud or negligence). And the second, damage or injury without the existence of a contractual antecedent, having origin in the action or omission from a merely civil view. Enshrined in article 1.902 of the code specifying the need for action or omission, causal link with proof of it, harmful result and guilt.

While criminal liability refers to punishable conduct or omissions committed fraud or negligence and defined as offences or misdemeanors in the Penal Code that may cause harm to patients during professional practice [11].

The Spanish code of ethics specifies the behaviors required of professionals, in the Obstetric-Gynecological Nursing practice. Internationally, the International Confederation of Midwives (ICM) establishes the midwife’s ethical obligations. These obligations include the relationship of midwives with other persons; the practice of midwifery; the fulfilment of their professional responsibilities and duties; and how they should work to ensure the integrity of the profession [12]. In Spain, it is regulated by the deontological regulations of the General Council of Nursing, however, due to the specific characteristics of the profession that distinguish it from the rest of general nurses, in 2011 the Spanish Association of Midwives produced a Code of Ethics for Spanish Midwives [13,14] to be able to protect the growing lawsuit and requirements from users of the professionalism of the people attending childbirth.

Informed consent (IC) has its origin in the Nuremberg Code of 1947 [15] and is regulated by Law 41/2002 of November 14, basic regulator of patient autonomy and of rights and obligations in matters of information and clinical documentation. In Spain, the Observatory on Obstetric Violence [16] defines obstetric violence as "ignorance of the emotional needs of the mother and the baby at any time of pregnancy, of childbirth and immediate postpartum, as well as of the authority and autonomy that women have over their sexuality, her body and her babies and the postures, rhythms and times that childbirth requires to develop normally". This implies that the midwife as a professional involved in the childbirth process could incur a civil crime for not providing health care according to *lex artis*.

Complaints constitute one of the most recurrent pillars for bringing civil liability claims against health workers, in the field of Obstetric-Gynecological Nursing where it plays a relevant role in accusations of obstetric violence in which the rights of IC and autonomy in the process of caring for pregnant women during pregnancy and childbirth are violated. These responsibilities are claimed by the users for reflecting the injuries of the fetus under the protection of Organic Law 10/1995, of 23 November, Book II Title IV called From Injuries to the Fetus [17].

Therefore, it is necessary to be able to analyze the content of the sentences, considering the motivation, their nature, as well as the sentences directed against the midwives.

### Objective

To evaluate judgments of the High Court of the Autonomous Community of Andalusia related to the professional practice of Obstetric-Gynecological Nursing.

## Materials and methods

### Identification of studies

The search for sentences was carried out through the jurisprudence database of the judicial documentation center (CENDOJ) [18] belonging to the General Council of the Judiciary (CGPJ), selecting as the judicial organ in the first place the Provincial Court of Seville turning scarce results, so it was decided to extend the search to the High Court of Justice of the autonomous community of Andalusia, considering itself the most appropriate. The keyword "midwife" was included as the search text and was temporarily limited from January 01, 2010, to the present day (last consulted November 17, 2022). The time criterion allows to establish a wide range in the selection of sentences to avoid the loss of those currently in judicial process and to try, in this way, to follow up on future appeals.

### Inclusion criteria

The inclusion/exclusion criteria are shown in Table 2.

**Table 2:** Inclusion/Exclusion criteria. Own elaboration.

Filters	Inclusion criteria	Exclusion criteria
Related terms	Midwife, obstetric-gynecological nurse	Gynecologist, tochologist
Publication Date	Last 12 years (2010–2022)	Preceding the year 2010
Languages	English, Spanish and Portuguese	Different Languages
Article Access	Full text access	Access to Summary
Databases	Free	Private (requires registration and payment)

The search for bibliography used for the introduction and discussion of this work was carried out through the main databases (Dialnet, Cuiden, Pubmed, Scopus) using the keywords "midwife," "civil liability," "informed consent," "competencies," "code of ethics," "obstetrics" and their translations into official foreign language through Medical Subject Heading terms (MeSH) and official legal information pages to complete the legislative perspective. (Table 3) The eligibility criteria, as well as the sources and diagnostic process of the selected articles and judgments were based on the STROBE Statement for observational studies.

**Table 3:** Descriptors DeCs y MeSH. Own elaboration.

TERMS DECS	TERMS MESH
Matrona	*Midwifery*, *nurse midwives*
Obstetricia	*Obstetrics*
Responsabilidad legal	*Liability*, *legal*
Consentimiento informado	*Informed Consent*
Competencia	*Professional competence*
Teoría Ética (Código deontológico)	Ethical Theory (Deontological Ethic)

Denial of access to certain databases for requiring prior payment will be a significant bias in our research study, because it will exclude several articles or judgments that would be valid in terms of content and relevance to our research question.

In relation to the search strategy, the Health Sciences Descriptors (DeCS) and the MeSHs related to the study topic will be used. Table 4 details the descriptors used and the order in the search following the PICO methodology:

**Table 4:** Search strategy health sciences descriptors/medical subject heading. Own elaboration.

	DeCS		MESH	
**P**	Matrona Obstetricia	OR	*Midwifery*, *nurse midwives* *Obstetrics*	AND
**I**	Responsabilidad legal Consentimiento informado Competencia Teoría Ética (Código deontológico)	OR	*Liability*, *legal* *Informed Consent* *Professional competence* *Ethical Theory (Deontological Ethic)*	AND
**C**	Do not apply the intervention			AND
**O**	Complicaciones del Embarazo Actividad profesional (profesionalismo) Seguridad del paciente	OR	*Pregnancy Complications* *Professionalism* *Patient Safety*	

## Results

**Fig 1:**
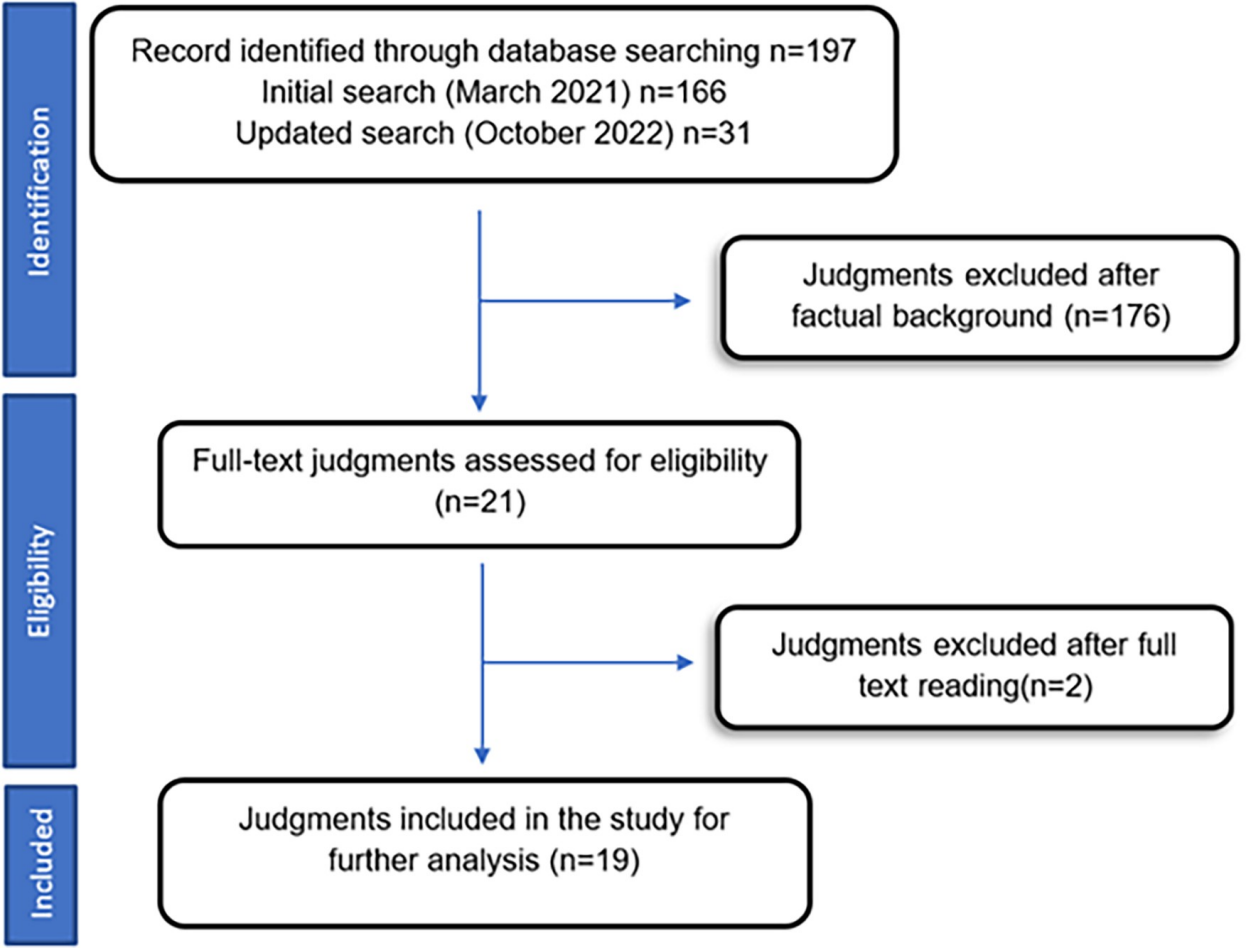
Flow chart of selected judgments. Own elaboration.

The preliminary search in the CENDOJ database yielded 197 results, of which 21 that were directly related to the topic studied were preselected, after reading in full text they were finally reduced to a total of 19 that will be analyzed later (Fig 1).

S1 File of the annex includes the main characteristics of the statements analyzed, in chronological order of publication.

A total of 19 judgements related to direct or indirect accusations against nurses specializing in obstetrics and gynecology were studied, of which 53% of judgments ended with a decision dismissing when the claim was made by the affected user, 21% of the accusations were partially upheld, 10% were upheld and the remaining 16% were dismissed for the Andalusian Health Service (SAS), considering the upheld for affectation for the user to be evidential.

**Fig 2:**
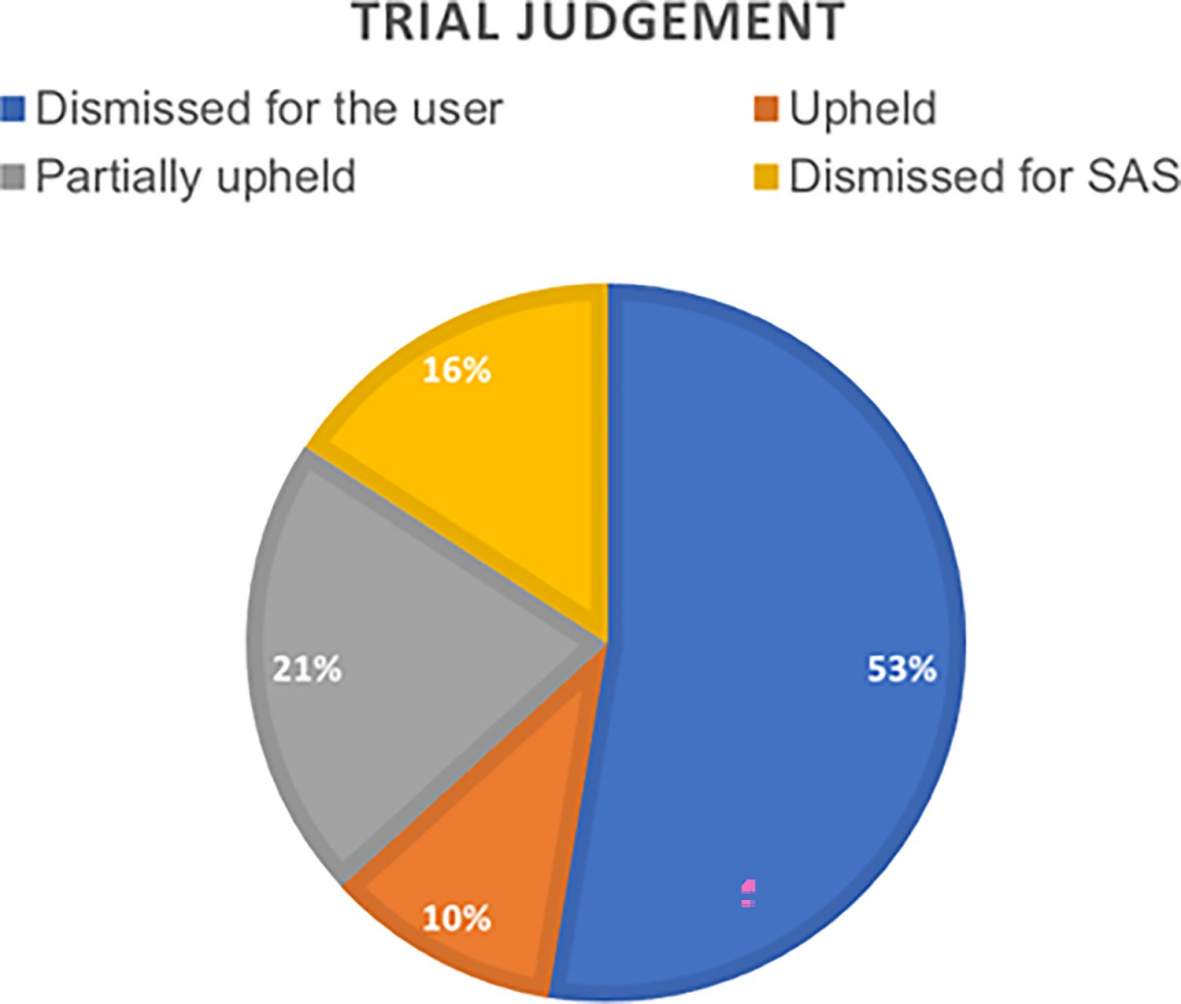
Judgments classified according to the ruling of the TSJ of Andalusia. Own elaboration.

The numbers of cases obtained in percentages are shown in Fig 2.

All the judgments analyzed derive from the contentious-administrative route, since the medical actions are directed against the administration as public service officials. Similarly, we can analyze that the accusations come from breaches of the *lex artis ad hoc* in relation to malpractice during childbirth care.

**Fig 3:**
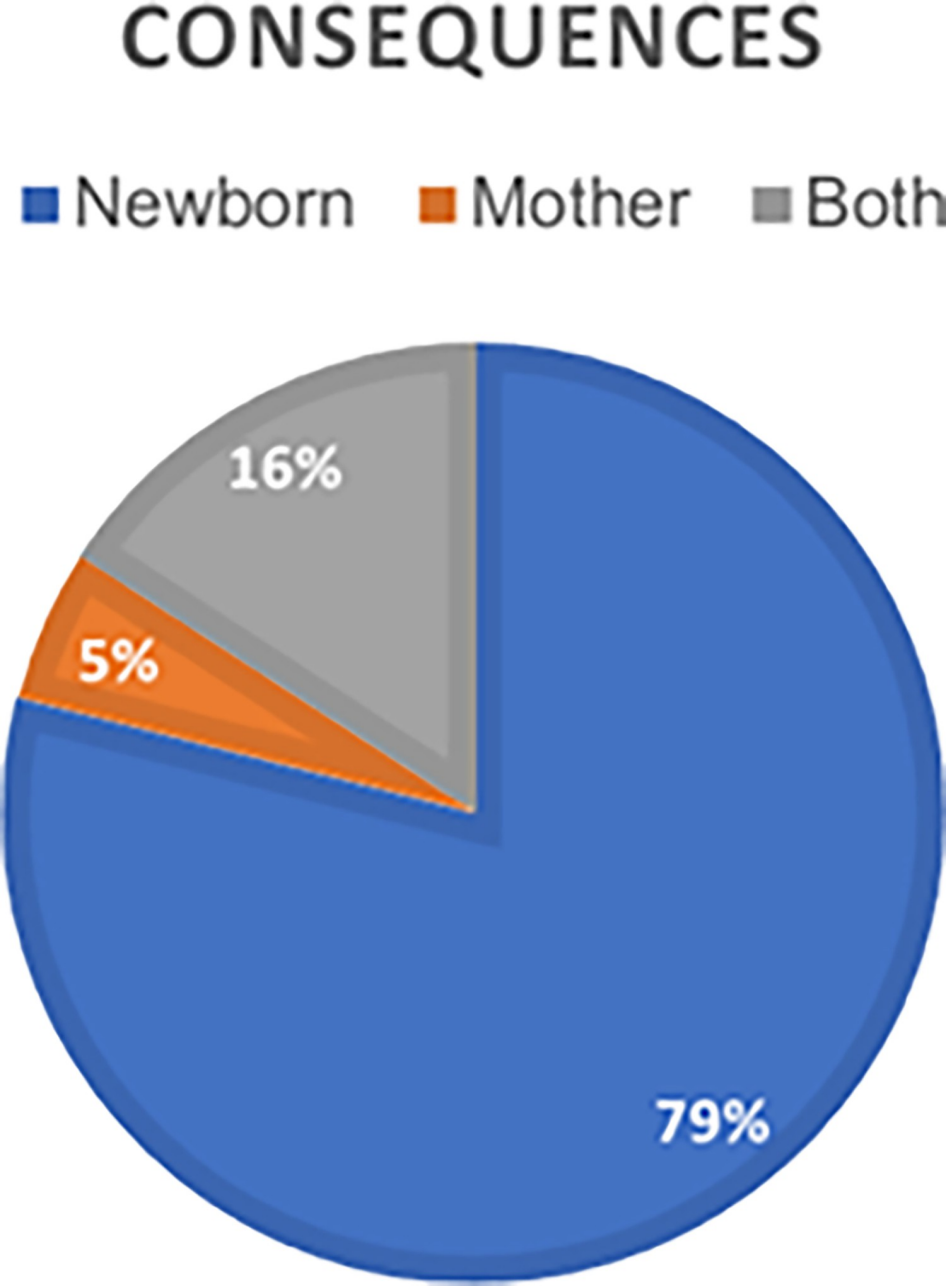
Judgments classified according to the consequences of the TSJ of Andalusia. Own elaboration.

The consequences of the proceedings have been divided into consequences for the newborn and the mother, these being subsequently classified and evaluated. Most of the consequences for which judicial accusations are those that affect the life of the newborn, occupying 79% of the sentences analyzed. Subsequently, 16% of the judgments are based on consequences for both and relegated to last place, with 5% those that are only directly related to the mother. The percentages obtained can be evidenced in Fig 3.

**Fig 4:**
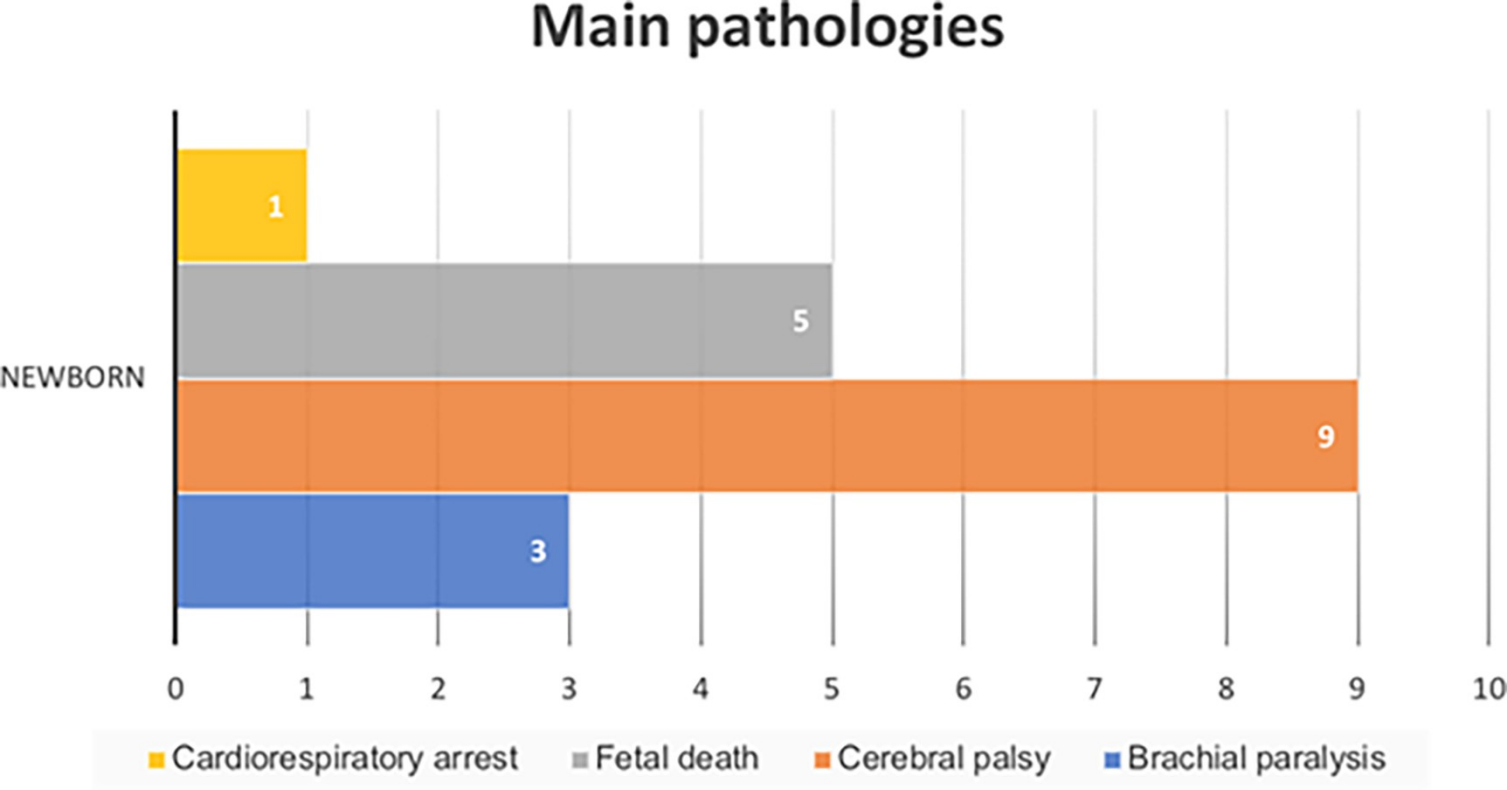
Judgments classified according to the main pathologies in the newborn TSJ of Andalusia. Own elaboration.

Subsequently, it will be established within the consequences that are the main pathologies or outcomes that resulted from the accusations studied. Cerebral palsy due to intrapartum cerebral hypoxia is the one with the highest number of accusations that have resulted in a study of professional exercise during obstetric care, followed by fetal death, brachial paralysis, and cardiorespiratory arrest (Fig 4).

**Fig 5:**
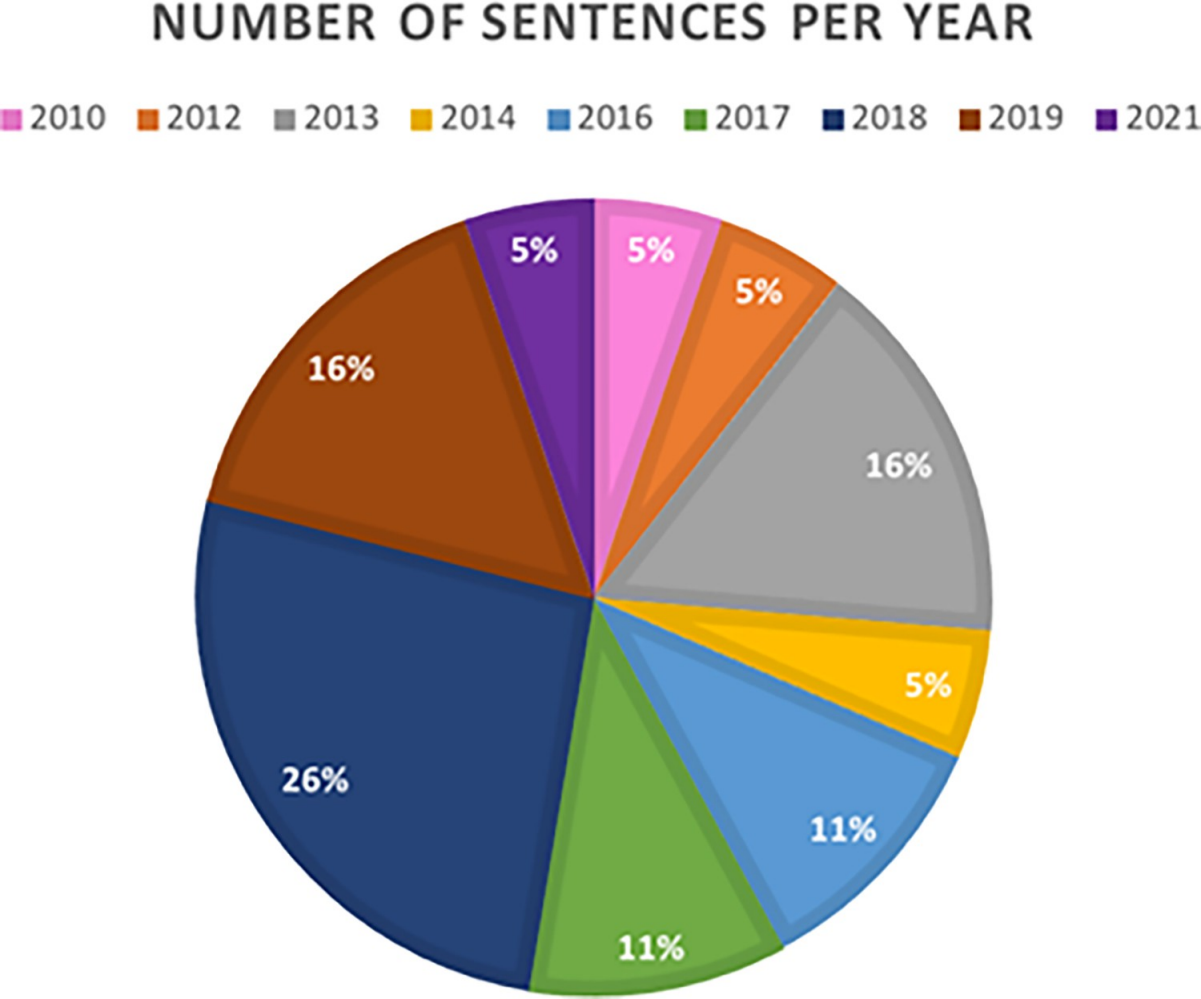
Sentences classified according to the year of the TSJ of Andalusia. Own elaboration.

Finally, the ranking of the number of sentences analyzed according to the year shows that 2018 is the first with 26%, followed by 2019 (16%) and 2013 (16%), then 2017 and 2016 both with 11%, and finally, the years 2010, 2012, 2014 and 2021 (5%). This data is shown in the figure below (Fig 5).

The decrease in the number of cases in 2021 may be due to the health crisis resulting from the Covid-19. This crisis has led to the adoption of a series of measures with high impact on the functioning of the Administration of Justice and, consequently, on the Contentious-Administrative Jurisdiction. Some of these measures result directly from the declaration of the state of alarm. Others are adopted later, during the period of transition to the new reality. Of all the measures adopted by the Government, the most significant has been the suspension of the procedural terms and times contained in the second additional provision of Royal Decree 463/2020 of 14 March, declaring the state of alarm.

Because of the measures taken by the Government during the state of alarm and because of the sanitary measures subsequently adopted by the governmental authorities to curb the Covid-19, an increase in litigation is very likely, resulting from the health crisis itself and the socio-economic impact of the measures implemented. Hence, an intensification of the already endemic collapse that afflicts the Contentious-Administrative Jurisdiction is easily foreseeable [19].

## Discussion

### Acquittal and conviction sentences

The study of the sentences analyzed with direct or indirect accusations about the Specialist Nurses in Obstetrics and Gynecology, we can see that most of the judgments (53%) ended with a decision dismissing when the lawsuit was made by the affected user, with the remaining 47% estimatory to varying degrees. The analysis of the sentences found shows that nurse specialists in obstetrics and gynecology receives most of the accusations of malpractice during childbirth care. Midwives are independent professionals from a scientific and legal point of view [20], so actions in women and newborns depend to a large extent on decisions made individually during obstetric care.

The analysis of acquittals sentences shows that the causes evidencing their dismissal were mostly that the actions and interventions were justified to reduce the risk to the mother and newborn, so the performance of the healthcare professional, in this case the midwife, was always in line with the *lex artis ad hoc*.

With a greater emphasis on the sentences analyzed, we can see that the fact of the loss of opportunity is named by concurrent circumstances. In the sentence, the midwife was attending other births, so she did not recognize the risk situation until it was too late, which ultimately caused the fetal death. This circumstance reveals the low midwife-woman ratio present in our country, if we analyze the data provided by the Ministry of Health in its " Informe sobre profesionales de enfermería. Oferta-Necesidad 2010–2025 "[21], we can corroborate that Andalusia, together with the Community of Madrid and Asturias, is at the tail of the specialized care in Obstetric-Gynecological Nursing, with the lowest midwifery ratios per 100,000 inhabitants, of just 22.9 at national level and 19.5 at regional level in Andalusia, this being lower than the average for OCDE countries.

Examined chronologically, there is an upturn in those in 2018. This may be due to multiple reasons: either because the number of fetal deaths increased in those years or because of an increase in women’s awareness of their own body and the delivery process they wish to experience. We do not know the real reason for failing to follow up on the relevant judgments.

### Rationale for judicial judgments

**Fig 6:**
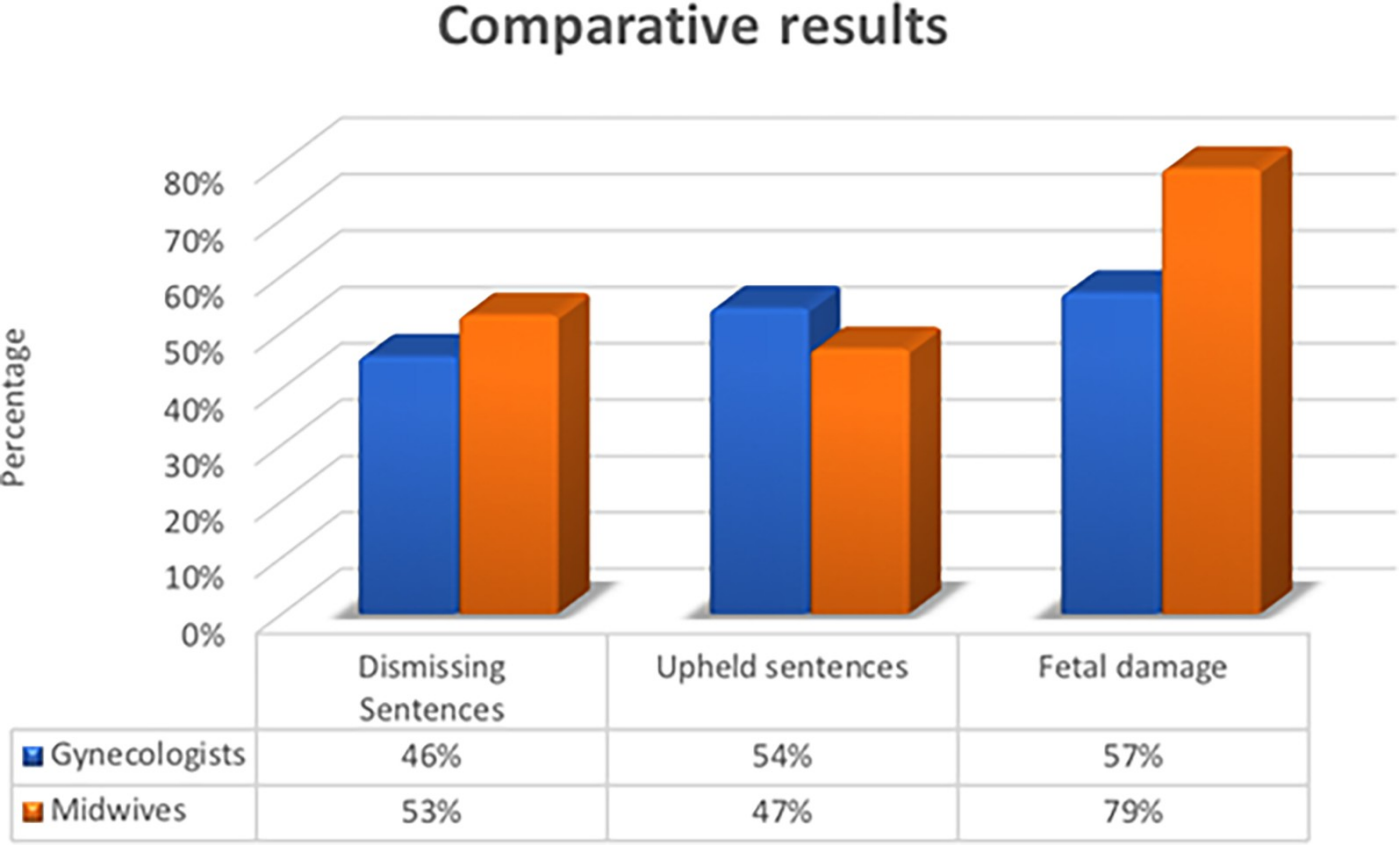
Comparative results between García-Ruiz N article and our investigation. Own elaboration.

The article made by García-Ruiz N. [10] in 2017 evaluate judgments against obstetricians and gynecologists, we can see some coincidence with the results obtained in our study. This article shows that 57% of the events that were convicted were related to fetal harm or death (Fig 6). Our analysis has turned similar results in this sense, because the processes with the highest number of demands have been those in which the newborn had signs of hypoxic-ischemic encephalopathy (cerebral palsy), followed by fetal death. If we study in greater detail the estimatory or partially estimatory convictions, we found that 6 of the 9 failures were related to brain injuries that affected to a greater or lesser extent the life of the newborns, causing serious consequences such as varying disabilities, spasms, epileptic crises, and psychomotor delay.

This article also analyzes the fact that mothers have risks in obstetric care. The sentences analyzed show that complaints of maternal injuries represent 5%, obtaining all dismissal decisions. It should be noted that only in cases where the affectation was the mother-newborn binomial, we find estimatory judgements. In relation to this topic, it is desirable to bear in mind the existence of obstetric violence [16] in the midwifery of our country, these practices violate the right of women to autonomy over their own bodies in the process of childbirth, infantilizing them and relegating them to positions of submission.

The sentences analyzed show on 2 occasions the realization of a maneuver to accelerate the extraction of the fetus, called Kristeller. This technique was developed to avoid the use of obstetric instruments (forceps or suction cup) during the second stage of childbirth. In recent scientific publications, it has been shown that the performance of Kristeller’s maneuver [22] can cause damage to the mother, such as uterine rupture, genital prolapse, perineal lacerations, sphincter damage, and higher episodic ratio. The newborn is not exempt from danger by performing this practice but increases the risk of brain damage and brachial plexus injuries. In Spain, the percentage of post-partum women who had performed the above-mentioned maneuver amounted to 69.2% of the cases [23]. From the legal point of view, Law 41/2002 of 14 November, patient autonomy legislates the importance of informing women of all procedures to be carried out and seeking IC, in contrast, 93.5% of women do not receive such information regarding the consequences of such intervention, violating bioethical principles (autonomy and non-maleficence). Of relevance is the fact that these proceedings have not yet gone beyond the legal sphere, as we can see, none of the sentences analyzed has been considered by this fact, the existing precedents of other judicial decisions in autonomous communities such as Valencia, Galicia, Extremadura or Castile and León [24].

Another interesting article was the one made by Bolcato M. [25], that presents the findings of an analysis conducted on medical professional liability disputes filed at a Level III University Hospital in Italy between January 1, 2003, and December 31, 2019. During the specified period, the Medico-Legal Services archives contained a total of 1451 cases. Out of these cases, 130 claims were specifically related to obstetrics-gynecology, accounting for 9% of the total number of cases. This department ranked third in terms of the highest number of claims, with general surgery and orthopedics taking the top two positions. Among the obstetrics-gynecology claims, 29% involved damages sustained by infants or unborn children, while the remaining 71% were related to adult female patients. In terms of the healthcare professionals involved in these cases, 67% were consultant doctors, 19% were resident physicians, and 14% were healthcare professionals, particularly midwives.

In the obstetrics-gynecology area, 40% of the compensation claims were accepted. This acceptance rate is higher than the overall average acceptance rate documented in the medico-legal watchdog database for the same period, which was 33%.

### Contentious-administrative judgments

First, we must point out the fact that our work was not limited to the civil way, however, the whole of the sentences analyzed belong to contentious-administrative procedures, the ultimate purpose of these accusations being monetary compensation and not the punishment of the professional through disqualification or custodial sentences. The costs generated to cover the compensation arising from these processes are among the highest in the health professions [26,27]. The economic burden borne by hospitals, private clinics, and professionals results in the payment of private liability insurance to ensure that the damage caused in most cases in the newborn is compensated for the serious permanent consequences considered. The national statistical institute records that in the year 2020 the 24% of births with a single fetus were performed through cesarean section, reaching 25.37% in the autonomous community of Andalusia. WHO emphasizes that caesarean section rates of more than 10% are not related to reductions in maternal or neonatal mortality [28], therefore, the implementation of these interventional measures does not ensure the absence of harm in the newborn.

The processes studied highlight on 7 occasions (36%) the defects in the Medical history (MH) necessary to understand the events that occurred during childbirth, mainly, the CTG and partogram. The desirability of attaching these documents to the judicial process lies in the justification of obstetric conduct. The MH is positioned as a transcendent instrument in the legal field, although its regulation is dispersed and incomplete. This is due to the incoordination existing in political matters that allows the decentralization of the Autonomous Communities in the exercise of competences and regulation in the minimum data set. The midwife issues reports expressing evaluations and analysis of the assistance provided from a subjective point of view, relying on the objective part of the expert report that analyzes the data based on evidence or studies, trying to understand the situations in which the different actions have been carried out. For all this, the MH is a document with legal implications that brings together evidence that shows possible negligence or malpractice, and, consequently, must contain all the data that may be accurate in the process of childbirth care, for example, medication, tests performed, diagnoses, complications… collected mostly in the partogram.

In the same way, the Clinical History should contain the CTG performed on the woman during her hospital stay that allows to assess the variability or reactivity of the fetus during obstetric care at childbirth. The main limitation of the CTG lies in its subjectivity of interpretation, with the professional having to evaluate and decide accordingly. The observer’s bias has been tried to overcome with new protocols that allow to decrease the inter and intra-observational variability, however, there is no consensus and for now it is up to the midwife to assess the fetal well-being.

In judicial terms to be able to talk about fetal asphyxia, 3 fundamental requirements must be met: acidotic pH, low-score Apgar test and neurological sequelae. Having reviewed the judgments, we can verify that on 5 cases request the fetal pH test as a biochemical marker to check the relationship between a CTG non-tranquilizing tracing and fetal hypoxia suspicion. Such a pH test may be performed intrapartum by a microtome in or after the fetal scalp, through the umbilical cord sample of the newborn. The article of East CE et al. [29] carried out in 2015 on the pH of fetal scalp, showed that there are no significant differences in the results expected in relation to the decrease of adverse perinatal events, in contrast, this procedure would increase the number of implanted births and therefore the risks would outweigh the benefits.

Within this order of ideas, another of the most important components required in court rulings is the clinical marker of the Apgar test that evaluates the newborn at birth, at 5 minutes of life and in exceptional cases at 10 minutes. The test consists of 5 factors: appearance, pulse, reflexes, activity, or tone and breathing effort; these factors will be scored from 0 to 2, being 10 the maximum score obtained. This information is useful to relate whether hypoxia has occurred during the delivery process or later. The Spanish Association of Pediatrics classifies an Apgar between 0–6 after 5 minutes of life as a non-specific criterion but suggesting the presence of an adverse perinatal event related to asphyxia episode causing brain damage and subsequent neurological sequels [30].

These evidentiary elements gain special relevance in cases of reversal of the burden of proof, to allow the exemption of the healthcare professional. Childbirth is not a risk-free process, in most cases it occurs in a physiological way and has no greater connotations for the life of the mother or newborn, but at certain times this process can lead to injuries in both.

A collection of cases on the management of clinical risk can provide valuable opportunities for midwives in several ways. Some potential benefits could be:

1. Learning from real-life scenarios: Case studies allow midwives to explore and analyze actual situations where clinical risks were encountered. By examining these cases, midwives can gain insights into the complexities and challenges of managing clinical risks in a practical context.
2. Enhancing decision-making skills: Reviewing a collection of cases can help midwives develop their critical thinking and decision-making abilities. They can analyze different risk management strategies employed in various scenarios, understand the rationale behind decisions made, and identify effective approaches to mitigate risks.
3. Developing risk assessment skills: Case studies provide an opportunity for midwives to practice risk assessment. By examining the factors contributing to adverse events or near-misses in different cases, midwives can learn to identify potential risks, assess their likelihood and potential impact, and implement preventive measures accordingly.
4. Improving communication and teamwork: Many cases involve multidisciplinary care teams, requiring effective communication and collaboration. Analyzing such cases can help midwives understand the importance of clear communication, teamwork, and coordination with other healthcare professionals to manage clinical risks effectively.
5. Promoting reflective practice: Reflecting on case studies enables midwives to engage in self-assessment and continuous professional development. They can critically evaluate their own practice, identify areas for improvement, and apply lessons learned from the cases to enhance their clinical skills and patient safety practices.
6. Sharing best practices: A collection of cases can be used as a platform for sharing best practices and lessons learned among midwives. Discussions and debriefings around the cases can facilitate knowledge exchange, fostering a culture of shared learning and continuous improvement within the midwifery community.
7. Promoting risk awareness and prevention: By examining cases where clinical risks have occurred, midwives can develop a heightened sense of risk awareness. This can contribute to proactive risk mitigation strategies, increased vigilance, and a culture of safety within their practice settings.

Overall, a collection of cases on the management of clinical risk offers midwives valuable opportunities to learn from real-world experiences, improve their risk management skills, enhance decision-making abilities, and ultimately provide safer care to their patients.

### Limitations of the study

The limitations of the present study lie, first, in the delay in obtaining a judicial decision. The Transparency Act stipulates the time to properly settle court judgements; in the specific case of the TSJ, in the civil field, the duration in months has been decreasing from 20.3 in 2010 to 13.8 in 2019, reached in Andalusia the average of 14.3 months in the course of 2019 [31]. This makes it difficult to be up to date with the decisions that are taking place this year and, consequently, to follow up on judgements to determine whether future appeals of the summits could vary from dismissal to dismissal.

Secondly, the affected users have a prescribed period of 1 year to file complaints in the case of medical negligence, beginning the calculation since the negligence occurs. However, in cases of injury or damage, this period begins to compute since they are consolidated as sequels, and we can know the definitive scope with reasonable prognosis. Therefore, if we add to this fact the time limits of judicial decisions, our study has a gap of about two years in the study of sentences.

Finally, our work has focused on the TSJ of the Autonomous Community of Andalusia, thus ignoring the judgments against Obstetric-Gynecological nurses that have occurred in other Autonomous Communities, or the consultation of highest judicial bodies such as the Supreme Court, National Audience, or the High Court of Justice.

### Conclusion

The present study evaluated 19 judicial judgements covering the period from 2010 to 2022, corresponding to the High Court of Justice of the Autonomous Community of Andalusia using the database of the General Council of the Judiciary (CENDOJ). After the hypotheses and objectives, we can conclude that:

1. The number of acquittal judgments exceeded in terms of percentages by 53%, to convictions that were established at 47% (21% of the accusations were partially upheld, 10% were upheld and the other remaining 16% were dismissed from the Andalusian Health Service considering the estimation by affectation for the user to be evidential).
2. The 79% of the court claims were filed for damage to the newborn, including frequent and severe cerebral palsy and intrapartum fetal death.
3. All judicial complaints studied in the field of Obstetric-Gynecological Nursing are resolved through the contentious-administrative route, because of breaches of the *lex artis ad hoc* in relation to malpractice during childbirth care.
4. The 5% of the judicial sentences analyzed correspond to maternal injuries of different types: uterine rupture, genital prolapse, perineal lacerations, damage to the sphincter.
5. Damage to the mother-child binomial represents 16% of the total number of judgments analyzed and, in this case, all of them were upheld by the relevant judicial body.
6. Knowledge of the reasons for judicial judgements in the field of Obstetric-Gynecological Nursing would constitute a convenient way to prevent possible errors in professional actions and, consequently, an improvement in the professional exercise and safety of the patient.
7. The analysis of the complaints in the Obstetric-Gynecological Nursing filed in the Provincial Hearings, TSJ of the rest of autonomies, as well as the appeals before the Supreme Court would constitute future lines of investigation that would be complete in a more exhaustive way the current study.

## Supporting information

S1 File Characteristics of revised sentences in chronological order of publication (2010–2021). (DOCX)
