# Supplementary material for: Professional responsibility in obstetric-gynecological nursing: Update of the case law of the High Court of Justice of Andalusia (Spain)
Source: PLoS One. 2023 Sep 26;18(9):e0291669. doi: 10.1371/journal.pone.0291669 (PMC10521977; doi:10.1371/journal.pone.0291669)
Supplement: S1 File — (DOCX) [file pone.0291669.s001.docx]

**Supporting information**

**General hypothesis**

The analysis of the judicial claims of the High Court of Justice (TSJ) of the Autonomous Community of Andalusia in the field of obstetric-gynecological nursing, will bring benefits for the improvement of the healthcare activity in relation to the professional exercise of midwives and the safety of the patient, and the mother-child binomial.

**Specific hypothesis**

1. The study of the decisions of the TSJ of the Autonomous Community of Andalusia will obtain a higher percentage of absolute judgments than convictions for the midwife defendants.
2. The study of court rulings will find a greater relevance and frequency of cases in which injuries occur on the newborn.
3. The investigation concerning the judicial (civil, criminal, and contentious-administrative) order of the judgments will gather information to catalogue the litigations and know the law applied.

**Specific Objectives**

1. Determine the number of acquittals and convictions in terms of percentage of the decisions of the TSJ of the Autonomous Community of Andalusia related to the professional performance of the midwives.
2. To analyze the motivation of the judicial judgments of the TSJ of the Autonomous Community of Andalusia related to the professional performance of the midwives.
3. Examine the jurisdictional order applied in the judgments of the TSJ of the Autonomous Community of Andalusia with professional performance of the midwives: administrative, criminal, or civil litigation.

| **S1 Table 5**. Characteristics of revised sentences in chronological order of publication (2010-2021) | | | | | | |
| --- | --- | --- | --- | --- | --- | --- |
| NUMBER OF PROCEDURE | PROCEDURE | ACUSSATION | CONSECUENCES | JUDGMENT | JUSTIFICATION |  |
| 502/2009  Date 11/05/2010 | Contentious-administrative | Malpractice in childbirth care | For the Newborn: Hypoxic-ischemic syndrome and perinatal asphyxia  Long-term: Tetraparesia, epilepsy. | Dismissed | There are no objective reports on the extent of the aftermath.  Reached the deadline of 1 year to file an appeal. |  |
| 220/2011  Date 10/09/2012 | Contentious-administrative | Malpractice in childbirth care   - Presence of midwife without doctor - No reported consent (IC) for vaginal delivery with risk factors | For the Newborn:  Shoulder dystocia and possible brachial paralysis | Dismissed | Health care has been correct during childbirth, according to current protocols.  The user cannot choose between vaginal delivery or cesarean section. |  |
| 444/2011  Date 03/11/2013 | Contentious-administrative | Malpractice in childbirth care:   - Wrong decision to have delivered a premature twin birth spontaneously - Loss of data recorded in the monitoring of pregnancy - Lack of custodial duty by the Civil Service | For the Newborn: Multicystic encephalomalacia  Long-term: variable psychomotor delay, spasticity, and epileptic seizures | Partial upheld | Fetal suffering due to loss of monitoring (CTG) data has not been proven. |  |
| NUMBER OF PROCEDURE | PROCEDURE | ACUSSATION | CONSECUENCES | JUDGMENT | JUSTIFICATION |  |
| 335/2012  Date: 06/14/2013 | Contentious-administrative | Neglect of health care:   - Lack of IC for induction into childbirth - There is no record in Medical History (MH) of medication and dosage administered - Kristeller - Episiotomy without mediating consent - Manual removal of placenta without waiting time - Counterfeit time of birth in MH | For the mother:  Maternal hemorrhage  Damage to the pelvic floor, vagina and bladder  Posterior anal fissure  In the long term:  Urinary incontinence  Interstitial cystitis  Nerve trauma rotting  Psychological damage and post-traumatic stress | Dismissed | Intervention actions were justified to reduce the risk to the mother and newborn.  The performance was always in line with the *lex artis.*  Subjective assessment not performed by an expert, lacks technical-medical support.  Consent is always provided verbally because childbirth is considered a physiological process. |  |
| 445/2011  Date: 07/25/2013 | Contentious-administrative | Malpractice in childbirth care resulting from the breach of the duty to foresee danger to the fetus because of the presence of obvious signs of fetal discomfort:   - Lack of CTG for 17 min due to loss of fetal focus - 51-minute delay in cesarean section practice | For the Newborn:  Fetal Death | Dismissed for SAS | Incorrect action in violating the duty of registration and clinical documentation that entails the deprivation of accreditation of the relationship between malpractice and fetal death. |  |
| NUMBER OF PROCEDURE | PROCEDURE | ACUSSATION | CONSECUENCES | JUDGMENT | JUSTIFICATION |  |
| 45/2011  Date 03/11/2014 | Contentious-administrative | Malpractice for violation of the duty to foresee danger to the fetus because of the presence of obvious signs of fetal discomfort:   - Delay in performing the cesarean section - Lack of CTG graphics | For the Newborn:  Cerebral palsy of type spastic tetraplegia | Partial SAS upheld for payment reduction | Loss of opportunity to prove the causal relationship of a malpractice and the neurological damage of the child. |  |
| 9/2015  Date 01/20/2016 | Contentious-administrative | Malpractice in the application of epidural technique:   - Coercion by the midwife - No consent - Difficult puncture (hematic) | For the mother:  Mistreatment and humiliation  Cesarean section | Dismissed | There is no evidence that the use of epidural has resulted in cesarean section. |  |
| 951/2010  Date 12/22/2016 | Contentious-administrative | Malpractice in childbirth care due to loss of opportunity:   - 25-minute waiting time between fetal bradycardia and cesarean section - Lack of observation measures by the midwife | For the Newborn:  Death 22 days after birth from severe hypoxic-ischemic syndrome and sepsis by *S. Virians* | Dismissed | Healthcare professionals acted according to *lex artis.*  Competing circumstances: the midwife was attending to 3 more deliveries and the gynecologist was attending to an urgent cesarean section. |  |
| NUMBER OF PROCEDURE | PROCEDURE | ACUSSATION | CONSECUENCES | JUDGMENT | JUSTIFICATION |  |
| 304/2015  Date 10/09/2017 | Contentious-administrative | Infringement of *lex artis* due to failure to observe the risks of childbirth:   - Lack of special surveillance - Lack of control over the evolution of the fetus - Delay in cesarean section practice | For the mother: uterine rupture and bladder lesions  For the Newborn: Fetal Death | Partially Upheld | Mala praxis: 1 hour cesarean section delay was due to confusion of fetal heart rate with maternal heart rate |  |
| 498/2015  Date 10/23/2017 | Contentious-administrative | Malpractice in childbirth care:   - Meconium - Prolonged expulsion period - Birth attended by midwife who does not warn gynecologist | For the Newborn:  Intrapartum fetal hypoxia  Spastic-dystonic infantile cerebral palsy  Long-term: 89% disability | Dismissed | Malpractice during childbirth and subsequent care is not proven.  There was no cerebral palsy at birth. |  |
| 416/2015  Date 01/12/2018 | Contentious-administrative | Malpractice in childbirth care:   - Lack of CTG - Delay in the cesarean section | For the Newborn: Hypoxic Perinatal Encephalopathy | Dismissed | There is no cause-effect between reported lex artis violations and the consequences inherent in a hypoxic process.  There are no recorded objective signs of fetal discomfort. |  |
| NUMBER OF PROCEDURE | PROCEDURE | ACUSSATION | CONSECUENCES | JUDGMENT | JUSTIFICATION |  |
| 241/2017  Date 03/08/2018 | Contentious-administrative | Malpractice in childbirth care:   - No proper scans were performed in obstetric consultation - No Pose Test Performed - CTG interpretation error - Neglect to perform scans - Imprudence for not performing cesarean section after 31 minutes of pathological CTG | For the Newborn: Bradycardia and meconium with cardiorespiratory arrest  Long-term: 65% disability grade | Upheld | There is a direct cause-effect relationship between pre-natal fetal well-being control assistance and childbirth with the perinatal outcome. |  |
| 489/2017  Date 04/05/2018 | Contentious-administrative | Malpractice in childbirth care:  Kristeller maneuver in woman with a history of shoulder dystocia, gestational diabetes, and upheld fetal weight of 4000 kg | For the Newborn:  Brachial plexus lesions, collarbone fracture, intracranial bleeding, epilepsy, and hypoxia | Partially Upheld | The only sequel attributable to shoulder dystocia is brachial plexus injury, but not intracranial bleeding.  The user showed little collaboration not wanting to undergo monitoring and concealing her history of risk. |  |
| NUMBER OF PROCEDURE | PROCEDURE | ACUSSATION | CONSECUENCES | JUDGMENT | JUSTIFICATION |  |
| 1338/2017  Date 09/19/2018 | Contentious-administrative | Malpractice in childbirth care:   - Lack of constant attention and detailed follow-up - Incorrect interpretation of CTG - 15-minute decalage between the monitor and printed paper | For the mother: uterine rupture  For the Newborn: Fetal Death | Dismissed for SAS | Constant and detailed lack of follow-up in a situation of risk pregnancy due to previous cesarean section and difficult delivery. |  |
| 853/2017  Date 10/04/2018 | Contentious-administrative | Infringement of *lex artis* due to non-observance of alarm signs:   - Meconium - Presence of decelerations - Little cervical dilation | For the Newborn: perinatal asphyxiation and hypoxic-ischemic encephalopathy | Upheld | Violation of *lex artis* in childbirth care.  Essential criteria are met to consider that asphyxiation has caused brain damage. |  |
| 329/2017  Date 02/07/2019 | Contentious-administrative | Malpractice in childbirth care due to insufficient documentation generated:   - Blank partogram - Lack of evidence of any measures taken to ensure fetal well-being | For the Newborn: Fetal Death | Dismissed | There was no administrative silence.  Malpractice intervention in the resulting result should be considered proven. |  |
| NUMBER OF PROCEDURE | PROCEDURE | ACUSSATION | CONSECUENCES | JUDGMENT | JUSTIFICATION |  |
| 483/2017  Date: 05/21/2019 | Contentious-administrative | Malpractice in childbirth care due to risk factors indicating cesarean section: obesity, nulliparity, gestational diabetes and macrosomia | For the Newborn: Brachial paralysis due to shoulder dystocia | Dismissed | Correct presence of the doctors and midwives who attended the delivery, maneuvers carried out quickly and effectively. |  |
| 168/2017  Date: 07/15/2019 | Contentious-administrative | Malpractice in childbirth care:   - Meconium - Prolonged expulsion period - Birth attended by midwife who does not warn gynecologist | For the Newborn:  Intrapartum fetal hypoxia  Spastic-dystonic infantile cerebral palsy  Long-term: 89% disability | Dismissed | There is no substantial modification of the cause of malpractice (498/2015) |  |
| 667/2018  Date 02/2021/2021 | Contentious-administrative | Malpractice in childbirth care:   - Lack of matron annotation indicating fetal state alteration - Delay in performing cesarean section due to maternal risk factors (Hypertension) | For the Newborn:  Intrapartum fetal hypoxia with parenchymal hemorrhage | Dismissed for SAS | Absence due to misleading of documents and charts of childbirth  Inability to prove that the assistance provided met the requirements of the l*ex artis* |  |
